# Effect of periodontal treatment on the clinical parameters of patients with rheumatoid arthritis: study protocol of the randomized, controlled ESPERA trial

**DOI:** 10.1186/1745-6215-14-253

**Published:** 2013-08-14

**Authors:** Paul Monsarrat, Jean-Noël Vergnes, Alain Cantagrel, Nadège Algans, Sarah Cousty, Philippe Kémoun, Caroline Bertrand, Elise Arrivé, Christophe Bou, Cyril Sédarat, Thierry Schaeverbeke, Cathy Nabet, Michel Sixou

**Affiliations:** 1 Department of Dentistry, Toulouse University Hospital (CHU de Toulouse) and Toulouse Dental School, Paul Sabatier University, Toulouse, France; 2 Department of Rheumatology, Toulouse University Hospital (CHU de Toulouse), Toulouse, France; 3 Clinical Research and Innovation Department (DRCI), Toulouse University Hospital (CHU de Toulouse), Toulouse, France; 4 Department of Dentistry and Oral health, Bordeaux University Hospital, Bordeaux, France; 5 Department of Odontology, Bordeaux 2 University, Bordeaux, France; 6 Department of Rheumatology, Bordeaux University Hospital, Bordeaux 2 University, Bordeaux, France

**Keywords:** Rheumatoid arthritis, Periodontal diseases, Periodontitis, Randomized controlled trial, Protocol

## Abstract

**Background:**

Rheumatoid arthritis (RA) is a chronic inflammatory disorder that leads to joint damage, deformity, and pain. It affects approximately 1% of adults in developed countries. Periodontitis is a chronic oral infection, caused by inflammatory reactions to gram-negative anaerobic bacteria, and affecting about 35 to 50% of adults. If left untreated, periodontitis can lead to tooth loss. A significant association has been shown to exist between periodontitis and RA in observational studies. Some intervention studies have suggested that periodontal treatment can reduce serum inflammatory biomarkers such as C-reactive protein, or erythrocyte sedimentation rate. We hypothesize that periodontitis could be an aggravating factor in patients with RA, and that its treatment would improve RA outcomes. The aim of this clinical trial is to assess the effect of periodontal treatment on the biological and clinical parameters of patients with RA.

**Methods/design:**

The ESPERA (Experimental Study of Periodontitis and Rheumatoid Arthritis) study is an open-label, randomized, controlled trial. Subjects with both RA and periodontitis will be recruited at two university hospitals in southwestern France. In total, 40 subjects will be randomized into two arms (intervention and control groups), and will be followed up for 3 months. Intervention will consist of full-mouth supra-gingival and sub-gingival non-surgical scaling and root planing, followed by systemic antibiotic therapy, local antiseptics, and oral hygiene instructions. After the 3-month follow-up period, the same intervention will be applied to the subjects randomized to the control group.

The primary outcome will be change of in Disease Activity Score in 28 Joints (DAS28) at the end of the follow-up period. Secondary outcomes will be the percentages of subjects with 20%, 50%, and 70% improvement in disease according to the American College of Rheumatology criteria. Health-related quality of life assessments (the Health Assessment Questionnaire and the Geriatric Oral Health Assessment Index) will also be compared between the two groups.

**Discussion:**

Evidence-based management of potential aggravating factors in subjects with active RA could be of clinical importance, yet there are few randomized controlled trials on the effect of periodontal treatment on the clinical parameters of RA. The ESPERA trial is designed to determine if non-surgical periodontal treatment could improve clinical outcomes in patients with active RA, and the quality of life of these patients.

**Trial registration:**

The ESPERA Trial was registered in Current Controlled Trials [ISRCTN79186420] on 2012/03/20. The trial started recruiting on 2012/03/06.

## Background

Rheumatoid arthritis (RA) is a chronic and destructive systemic autoimmune disease, characterized by the accumulation and persistence of inflammatory infiltrates in the synovial membrane, and the inflammatory condition of joints, tendons, and periarticular structures [1,2]. If untreated, the inflammatory condition can lead to destruction of the bone and cartilage of joints, and also of ligaments and soft tissues, causing severe disability, clinically important impact on quality of life (QOL), and substantial effects in terms of cost and productivity loss [3]. RA affects approximately 0.3% of patients in France [4], with a usual female:male sex ratio of 3:1 [5].

Periodontal diseases are diseases that affect the tissues supporting the teeth. The first step in the gum-disease process is gingivitis, an immune-inflammatory response to the bacterial colonization of tooth surfaces, without bone loss. Gingivitis can progress into periodontitis, an advanced and more serious stage of gum disease, which includes alveolar bone loss, eventually leading to tooth loss [6]. Periodontitis is a chronic oral infection caused by inflammatory reactions to gram-negative anaerobic bacteria, and affecting about 35 to 50% of adults [7,8]. Several recent research studies have shown that periodontitis could have an important influence on systemic inflammatory loading, and could trigger or worsen many medical conditions, including myocardial infarction and stroke [9,10], unbalanced glycemic control in patients with diabetis [11,12], preterm births [13,14], occurrence of chronic obstructive pulmonary disease and respiratory complications [15], or even erectile dysfunction [16].

RA and periodontitis share some pathogenic features; both are chronic inflammatory diseases with genetic and environmental influences and immunoregulatory imbalance, and both lead to destruction of conjunctive and hard tissues [17,18]. A study has reported that the frequency of RA is significantly higher in patients with periodontal disease than in subjects without periodontitis (3.95% versus 0.66%) [17]. Other studies have reported a higher incidence of missing teeth, dental plaque, greater periodontal pocket depth, or worse clinical attachment levels in patients with RA [18-20]. On the one hand, it is likely that patients with RA may encounter more difficulties in achieving good oral health because of joint pain or functional limitation [20,21], while on the other hand, it is hypothesized that periodontitis could be a risk factor or an aggravating factor for RA. In particular, the role of *Porphyromonas gingivalis*, a well-known periodontopathogenic bacterium, has been widely highlighted, because this oral bacterium has an endogenous peptidylarginine deiminase enzyme [22]. This enzyme is responsible for citrullination of arginine residuals, which is one of the crucial first steps in the development of RA [23-25].

A few studies have shown that periodontal treatment might induce a significant decrease in erythrocyte sedimentation rate (ESR) or Disease Activity Scores in 28 joints (DAS28) [26-29]. Further prospective studies, especially randomized controlled trials, are needed to determine whether full management of periodontitis in patients with RA results in improved clinical or QOL outcomes. The aim of this study is to assess the effect of periodontal treatment on the parameters of RA.

## Methods

The recommendation of the Consolidated Standards of Reporting Trials (CONSORT) statement will be followed [30].

### General design

The ESPERA (Experimental Study of PEriodontitis and Rheumatoid Arthritis) trial is an open-labeled, randomized controlled clinical study. This trial is bicentric (Toulouse and Bordeaux University hospitals, France), with two parallel groups (‘immediate’ periodontal treatment versus ‘delayed’ periodontal treatment, with a balanced 1:1 treatment allocation), and with 3 months follow-up for both groups.

### Objectives

The primary objective of this trial is to analyze the hypothesis that periodontal treatment in subjects with RA will lead to a decrease in RA activity as, measured by DAS28 [31], after 3 months of follow-up in subjects suffering with both periodontitis and RA. The null hypothesis is that periodontal treatment does not reduce DAS28 in subjects with RA.

The secondary objectives are to assess whether periodontal treatment is associated with: 1) an improvement in RA, judged using American College of Rheumatology criteria (ACR) criteria (20%, 50% or 70% improvement; ACR 20, ACR50, or ACR70) [32]; 2), an increase in QOL as measured by the Health Assessment Questionnaire (HAQ) score [33,34]; and 3), an increase in oral QOL using the Geriatric Oral Health Assessment Index (GOHAI) score [35].

### Study participants

#### Selection criteria

Volunteers will be recruited from two Rheumatology Departments of southwestern France (Toulouse University Hospital and Bordeaux University Hospital). Patients who are referred to the either rheumatology department and have a positive diagnosis of RA (according to the 1987 revised American Rheumatism Association criteria for the classification of RA [36]) will be screened for possible inclusion. Pre-inclusion and exclusion criteria will be checked at V0, by rheumatologists in each center, using a standardized questionnaire.

### V0: screening visit

#### Pre-inclusion criteria

• Age 18 years or older (of either gender).

• Affiliated to a health insurance scheme.

• RA diagnosed at least 1 year before V0.

• DAS28 score between 3.2 and 5.1 during the month preceding V0.

• No change in medication, dose, or formulation in RA treatment during the 3 months preceding V0.

• Available for all study visits over 3 months (V1 to V4).

• Presence of at least six natural teeth.

• Ability to give written informed consent.

#### Exclusion criteria

• Planned hospitalization for pre-existing condition apart from RA within 3 months after V0.

• Risk of infection:

◦ Presence of one or more known infectious diseases (HIV, hepatitis, infectious mononucleosis).

◦ Known clinically important renal disease (creatinine clearance < 60 ml/min) or liver disease.

◦ Unbalanced diabetes.

◦ Known risk of endocarditis.

• Permanent pacemaker [37].

• Use of anti-thrombotic treatment (heparin, anti-vitamin K).

• Severe difficulties in understanding written and spoken French.

• Chronic disorder requiring chronic or intermittent use of antibiotics.

• Known hypersensitivity to chlorhexidine digluconate.

• Participation in another intervention study.

• Known contraindications to both amoxicillin and clindamycin.

• Known contraindications to dental local anesthetic.

• Pregnancy or lactation or intent to become pregnant.

### V1 - inclusion visit

#### Inclusion criteria

• No change relative to pre-inclusion criteria or exclusion criteria at V0.

• Periodontitis (at least one site with periodontal probing depth ≥ 4 mm and clinical attachment level ≥ 3 mm on at least four different teeth).

• Moderate RA activity according to European League Against Rheumatism (EULAR) criteria, defined as DAS28 of 3.2 to 5.1 [38].

• Provision of informed consent (after 1 week cooling-off period).

• No planned changes in medication, dose or formulation of RA treatment during the following 3 months. No change in disease modifying anti-rheumatic drug treatment will be allowed during the 3 months of the study duration. In cases of painful flare, (defined as an increase of more than 0.6 on the DAS28, or as a pain assessed as greater than 50 on a visual analog scale), use of analgesic (non-steroidal anti-inflammatory drug or at worst, morphine derivatives) or steroidal anti-inflammatory drug (cure in less than 6 days) will be allowed. If these are insufficient, then if the patient’s condition requires it, a treatment change can be established by medical staff. In such cases, patient will not be excluded from the study but it will be taken into account in the statistical analyses (see section on Statistical methods).

#### Exclusion criteria

A subject will not qualify for enrolment if she/he presents at least one of the following.

• Acute oral infection.

• Acute dental pain (including pulpitis).

• Suspicious oral mucosal lesion requiring further analysis.

• Severe oral inflammation unrelated to periodontal conditions.

• Iatrogenic fixed or removable prosthesis leading to severe mucosal inflammation.

• Need for immediate tooth extraction.

Hospital-based oral care will be offered to such subjects, and the re-inclusion into the trial will be re-evaluated after remission of symptoms or required delay.

#### Exclusion criteria (all stages)

Subjects will be excluded from the trial if they no longer wish to participate for any reason.

### Study timelines

Subjects will be invited to participate after a brief explanation provided by the rheumatologist at V0. If they agree, they will be given an information sheet and the informed consent document, so they can read them before the next visit. The study dentist will contact the subjects to make an appointment (V1) within 3 weeks in the same rheumatology center. During V1, subjects will receive more detailed information about periodontal diseases and, if they are still willing to participate, they will give their written consent. At this point, all subjects will have an appointment with the dentists to undergo periodontal treatment (V2) within 15 days after V1.

Subjects will then be randomly assigned to one of the groups and will be contacted by phone within 3 days to confirm or cancel the V2 appointment, depending on whether they belong to the immediate or delayed treatment group. Both groups will then be followed up for 3 months.

Subjects from the immediate treatment group will receive a control visit 3 weeks after V2 (V3) and a final visit at 3 months (V4).

Subjects from the delayed treatment group will also have the V4 appointment, at which they will receive the periodontal treatment and will also have a control visit (V5).

**Figure 1:**
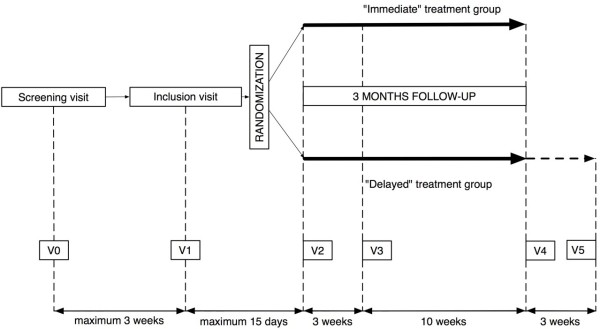
Study timelines.

Control visits will be used to check for proper healing of periodontal tissues and to repeat oral health hygiene instructions. The study timelines are presented in Figure 1 and details of study schedules are summarized in Table 1.

**Table 1:** Study schedule

	**V0**	**V1**	**V2**	**V3**	**V4**	**V5**
**“Immediate” treatment group**						
Review of pre-inclusion criteria	x					
Review of selection criteria		x				
Written informed consent		x				
Initial questionnaire		x				
DAS28 assessment		x			x	
ACR assessment		x			x	
HAQ and GOHAI self-questionnaires		x			x	
Full periodontal assessment		x			x	
Periodontal treatment			x			
Oral hygiene instructions			x	x		
Final questionnaire					x	
Next visit appointment	x	x	x	x		
**“Delayed” treatment group**						
Review of pre-inclusion criteria	x					
Review of selection criteria		x				
Written informed consent		x				
Initial questionnaire		x				
DAS28 assessment		x			x	
ACR assessment		x			x	
HAQ and GOHAI self-questionnaires		x			x	
Full periodontal assessment		x			x	
Periodontal treatment					x	
Oral hygiene instructions					x	x
Final questionnaire					x	
Next visit appointment	x	x			x	

### Randomization

The random allocation sequence will be generated using a computer number generator to select random permuted blocks. The block lengths will be 2, 4, 6 and 8 and will vary randomly. The random allocation sequence will be performed using R software (version 2.12.1) and the additional package ‘blockrand’ (version 1.2). Randomization cards will be printed out and sealed in sequentially numbered, opaque envelopes by an independent research assistant prior to the beginning of the trial. Randomization will be stratified by center. Subjects meeting the eligibility criteria at V1 will be randomly assigned to receive periodontal treatment within 15 days after V1 (immediate treatment group) or after the 3 months follow-up period (delayed treatment group (control)).

### Intervention

Periodontal treatment will be performed by trained and assessed dental investigators. Periodontal treatment is proposed as a comprehensive package including dental biofilm staining, full-mouth disinfection, non-surgical scaling and root planing, systemic antibiotic (ATB) therapy, and oral health hygiene instructions. The periodontal intervention will be performed at V2 for the immediate treatment group and at V4 for the delayed treatment (control) group. The delayed treatment group will receive neither periodontal treatment nor a placebo before V4. Finally, both groups will receive exactly the same intervention.

#### Scaling and root planing

A disclosing solution (Dento-Plaque Inava; Pierre Fabre, Gien, France) will be used to color the dental biofilm before starting the removal of supra-gingival and sub-gingival plaque and calculus. Scaling and root planing (SRP) will be performed using both hand instruments (Gracey curettes; Henry Schein, Alfortville, France) and an ultrasonic system. An ultrasonic generator (P-Max Satelec; Acteon, Mérignac, France) with specific micro instruments (H3, P2L, and P2R), using reduced power bands and a slight lateral pressure, will be used to perform mechanical debridement, under local anesthetic if necessary (in case of pain or on patient’s request). Chemical sub-gingival disinfection will be used along with the ultrasonic debridement, using an antiseptic mouth rinse (chlorhexidine 0.12%, Eludril Gé; Pierre Fabre).

#### Systemic (ATB) therapy

AFSSAPS (Agence Francaise de Securite Sanitaire des Produits de Sante (the French agency for the safety of health products)) and HAS (Haute Autorité de Santé (the French national health authority) recommend prophylactic ATB therapy for patients at risk of immunodeficiency undergoing periodontal treatment [39]. Sufficient amoxicillin (GlaxoSmithKline, Mayenne, France) for 7 days will be given to subjects on the day of the periodontal treatment. In case of contraindication to β-lactam ATBs, clindamycin (Pfizer, Paris, France) will be given. The detailed ATB therapy prescription will be adapted to each subject, based on 500 mg amoxicillin three times daily (morning, midday, and evening) for 7 days, or 300 mg clindamycin twice daily (morning and evening) for 7 days.

#### Oral hygiene instructions

Oral hygiene instruction (OHI) will be given by the dental investigators just after the periodontal treatment (that is, during V2 or V4, depending on randomization), and during V3 for the immediate treatment group or V5 for the delayed treatment group. A 15-minute oral session will include visual and verbal information on how to use a toothbrush, inter-space brushes, dental floss, and mouthwash correctly; how to clean bridges and dentures; and how and why to use a plaque disclosing test to perform tooth cleaning.

A full kit containing all the hygiene products necessary for 3 months of oral hygiene will be given to the patient at the end of the intervention. It will include three 75 ml tubes of chlorhexidine digluconate toothpaste (0.12%), 20 meters of dental floss, three soft toothbrushes, 12 single-tufted inter-space brushes, and a quantity of plaque disclosing tablets (Red-Cote; Gum Sunstar, Levallois Perret, France). A 300-ml bottle containing an antiseptic mouthwash product (chlorhexidine digluconate 0.12%, Sunstar Paroex; Chemineau Laboratories, Vouvray, France) will be given, with recommendations to use 10 ml of the product three times a day after tooth brushing for a period of 10 days after the periodontal treatment session. Leaflets with pictorial information, summarizing the various oral hygiene steps, will be included in the kit.

### Outcomes

The primary outcome is change in the DAS28 between V1 and V4. Secondary outcomes are difference in matching ACR20, ACR50, and ACR70 criteria, and changes in HAQ and GOHAI scores, between V1 and V4.

The assigned rheumatologist, who will be blinded to the patient’s assigned periodontal treatment group, will assess DAS28 [38], which is a continuous composite score (0 to 9.71) by considering the number of tender and swollen joints (out of the 28 evaluated joints); the ESR, which measures the degree of inflammation in the blood; and the patient’s global assessment of health. They will also assess the improvement in RA using the ACR criteria [32], and will review medical forms for each participant’s safety. The same rheumatologist will perform outcomes measures between V1 and V4. The health status of the enrolled subjects will also be assessed by the same rheumatologist throughout the monthly routine visits during the 3 months follow-up period. Interim or post-V4 data will be used in case of absence at V4.

Assessed dental investigators will be responsible for performing a standardized periodontal probing assessment at V1 and V4. The full-mouth periodontal probing will be performed using a constant pressure probe (Florida Probe^®^; Gainesville, Fl, USA), from six sites per tooth (mesiobuccal, mid-buccal, distobuccal, mesiolingual, mid-lingual and distolingual). Periodontal assessment will include measurement of periodontal pocket depth (PPD) in millimeters, clinical attachment level (CAL; in millimeters) and bleeding on probing (yes/no). No radiographs will be taken. Periodontitis will be defined by the presence of at least one site with PPD ≥ 4 mm and CAL ≥ 3 mm on at least 4 different teeth.

Finally, participants will self-complete questionnaires assessing QOL (HAQ and GOHAI). They will also be asked to complete initial and final questionnaires at V1 and V4, which will assess medical, social, and oral health behavior information, and assess changes during the 3-month follow-up period. Adverse events (AEs) during the follow-up period will be reported, and these will include unexpected study-related events and serious AEs regardless of cause.

### Statistical methods

Sample size calculation is based on detecting a difference of 0.6 points in DAS28 change from baseline between the two groups; 0.6 was chosen because it is the minimum therapeutic response according to EULAR [38]. Assuming a standard deviation of 0.6 [28] and using a two-sided test at the 5% significance level, enrolling 16 participants per group would yield 80% power (epiR package 0.9-30). Anticipating a 25% dropout rate, the target sample size was thus set at 40 participants.

After data cleaning, preliminary analyses will investigate the pattern of missing baseline and follow-up data. Baseline characteristics per group will be presented as mean, median, and standard deviations for quantitative variables, and as absolute and relative frequency for qualitative variables. Descriptive statistics will be provided for the intention-to-treat population to explore whether the randomization results in a balanced distribution of participants’ characteristics between the two groups.

All baseline analyses will be performed in accordance with intention-to-treat principles, regardless of the subject's presence at intermediate visits, the degree of compliance with the periodontal treatment, or any change in RA medication.

The main analysis will be a comparison between the two study groups concerning the change in the DAS28 scores from baseline to 3 months in a multivariate model, adjusted for clinical center, and considering the rest of the independent measures (including any change in RA medication) as possible modifying factors. The data will be secondarily analyzed using the ‘per protocol’ approach, in which the analysis will be restricted to participants who have complied with the treatment protocol (SRP, ATB, and OHI) and have had no changes in RA medication during study participation.

In addition to the primary analysis, five binary outcomes for DAS28 at 3 months (a relative decrease greater than 10%, a 3-month level of less than 3.2, an absolute decrease greater than 0.6, a relative increase greater than 10% and an absolute increase greater than 0.6) will be evaluated in logistic regression models. For secondary outcomes, continuous variables will be analyzed through analysis of covariance, using a model similar to that specified for the primary outcome.

Missing outcome data will be handled as described by Ware *et al.*[40] and Little *et al.*[41]. For participants who discontinue any of the periodontal treatments (SRP, ATB, or OHI) because of AEs or simple inconvenience, efforts will be made to obtain their consent for the collection of data on outcomes. Second, we will limit the burden and inconvenience of data collection on the participants, and make the study experience as positive as possible. For example, the DAS28 assessment will be performed in the clinical center where participants are usually followed for their RA treatment. Third, investigators will be trained that keeping participants in the trial until the end is important, regardless of whether they continue to receive the assigned treatment. This information will also be conveyed to study participants. Finally, missing outcome data will be handled using estimating-equation methods [42,43]. Complete cases will be weighted by the inverse of an estimate of the probability being observed. Efficacy of periodontal treatment will be assessed, as will the correlation between clinical response to periodontal treatment and DAS28 scores. The proportion of subjects who have one or more AEs in the two groups will be compared by Fisher's exact test. Analyses will be performed by R software (http://www.R-project.org).

### Ethics

The protocol and procedures have been approved by ethics and regulatory agencies, and will be implemented in accordance with the provisions of the Declaration of Helsinki [44]. The appropriate committee ((Comité de Protection des Personnes CPP (Committee for the Protection of Research Subjects), Sud-Ouest Outre-Mer I), approved the protocol on July 11, 2011 (number 1-10-37]). AFSSAPS approved it on November 4, 2010 (number 2010-A01193-36). The trial is registered on Current Controlled Trials under the number ISRCTN79186420 (http://www.controlled-trials.com/ISRCTN79186420/espera).

The informed consent form will contain the following information: names and affiliations of investigators, a plain language description of the study (treatment group, control group, and intervention), the duration of the study, the right to withdraw at any time, the ethics committee approval, and the privacy guarantee. All patients will receive free periodontal treatment (immediate or delayed), and will be fully informed of the potential AEs of periodontal treatment. The foreseeable risks are those conventionally attributed to periodontal treatment: temporary tooth sensitivity, temporary dental pain, side effects linked to oral antiseptics use, and side effects linked to oral ATB therapy.

## Discussion

Evidence-based management of potential aggravating factors in subjects with active RA could be of clinical importance. We hypothesize that periodontitis could be an aggravating factor for RA. However, there are few randomized controlled trials about the effects of periodontal treatment on the clinical parameters of RA. The ESPERA trial is designed to determine if non-surgical periodontal treatment could improve clinical outcomes in patients with active RA, and also improve their QOL.

Our trial has a small sample size, and a potential lack of power. However, the ESPERA trial has a conventional 80% power to detect a difference of 0.6 points in DAS28 change from baseline between the two groups, and 0.6 is the minimum therapeutic response according to EULAR [38]. We acknowledge that 0.6 mean improvement could be considered as relatively large, but two other trials have shown such an improvement [27,28]. Furthermore, the sample size corresponds to our source population of outpatients with both RA and periodontitis, as reported in a recently published observational study [45]. Because small trial size should not be used to justify low-quality trials, efforts will be made to minimize sources of bias, and to enhance the reliability of the results of the ESPERA trial. Selection bias will be prevented through randomization, and special attention will be paid to securing strict implementation of the random sequence. Performance bias will be unavoidable, because there is no possible placebo for periodontal treatment; however, detection bias will be prevented through the blinding of RA outcome assessors. Finally, attrition bias will be assessed at the end of the trial.

A strong feature of the current trial is that the expected benefit for the patient could be a contribution to the stabilization of the disease, or a slight clinical improvement in RA. This could reduce the need for escalating therapy related to insufficient control of the disease, and improve QOL in patients with RA. In long term, periodontal treatment could be integrated in the rheumatologist’s associated therapeutic arsenal.

Results of this trial could have an influence on clinical practice, and potentially contribute to further development of new research directions for both odontologists and rheumatologists.

## Trial status

The first patient was enrolled on March 6, 2012. The study is ongoing, and thus far has enrolled nine patients.

## Abbreviations

ACR: American college of rheumatology; AFSSAPS: French agency for the safety of health products; ATB: Systemic antibiotherapy; BOP: Bleeding on probing; CAL: Clinical attachment level; DAS28: Disease activity score 28; GOHAI: Geriatric oral health assessment index; HAQ: Health assessment questionnaire; HAS: French national authority for health; OHI: Oral hygiene instructions; PPD: Probing pocket depth; RA: Rheumatoid arthritis; SRP: Scaling and root planning.

## Competing interests

PM, JNV, CN, AC, and MS are working on a Cochrane systematic review ‘Interventions for periodontal disease in reducing the severity of rheumatoid arthritis’. Protocol published on September 12, 2012 (Vergnes J-N, Monsarrat P, Blaizot A, Nabet C, Cantagrel A, Sixou M, Furness S. Interventions for periodontal disease in people with rheumatoid arthritis. Cochrane Database of Systematic Reviews 2012, **9**: DOI:10.1002/14651858.CD010040).

## Authors’ contributions

PM and JV participated in the conception of the study and its design, and will perform statistical analyses; AC participated in the design of the study and the intervention, and has participated in reviewing the manuscript; NA participated in the conception of the study and its design; SC will participate in performing the treatment; PK was involved in drafting the manuscript and writing it, and will participate in analysis and interpretation of data; CBE was involved in drafting the manuscript and writing it, and will participate in performing the treatments; EA and CS will participate in performing the treatment; CBO participated in the conception of the study and its design; TS participated in the design of the study and the intervention, and has participated in reviewing the manuscript; CN has participated in reviewing the manuscript and will participate in analysis and interpretation of data; MS has participated in reviewing the manuscript, and will participate in analysis and interpretation of data. All authors read and approved the final manuscript.

## The ESPERA (Experimental Study on Periodontitis and Rheumatoid Arthritis) study group

The group consists of: Nadège Algans, Elise Arrivé, Thomas Barnetche, Caroline Bertrand, Christophe Bou, Alain Cantagrel, Marie-Hélène Cerato, Sarah Cousty, Gabriel Fernandez de Grado, Philippe Kémoun, Paul Monsarrat, Cathy Nabet, Delphine Nigon, Thierry Schaeverbeke, Cyril Sédarat, Michel Sixou, Delphine Thuillez, Jean-Noël Vergnes, and Claire Willmann.
